# The complete plastid genome of *Citrus hystrix* DC. 1813 (Rutaceae) and its phylogenetic analysis

**DOI:** 10.1080/23802359.2025.2449723

**Published:** 2025-01-07

**Authors:** Wan Shuan Lee, Warut Donrung, Bui Manh Hung, Nurien Hidayu Muhamad Rusly, Shiou Yih Lee, Tawatchai Tanee

**Affiliations:** a Department of Education and Liberal Arts, INTI International University, Nilai, Negeri Sembilan, Malaysia; b Centre of Health, Well-Being, and Environmental Sustainability, INTI International University, Nilai, Negeri Sembilan, Malayisa; c Department of Environment and Resource Studies, Mahasarakham University, Maha Sarakham, Thailand; d Department of Forest Inventory and Planning, Vietnam National University of Forestry, Hanoi, Vietnam; e Department of Health and Life Sciences, INTI International University, Nilai, Negeri Sembilan, Malaysia; f One Health Research Unit, Mahasarakham University, Maha Sarakham, Thailand

**Keywords:** Aurantidioideae, *Citrus micrantha*, genetic resources, kaffir lime, limau purut

## Abstract

The complete plastome size of *Citrus hystrix* DC. 1813 was 159,893 bp in length and has a typical quadripartite structure. The 87,148-bp-long large single-copy and the 18,763-bp-long small single-copy regions were separated by a pair of inverted repeats (each 26,991 bp). The plastome was predicted to contain 132 genes, of which 87 were CDS, 37 were tRNA, and eight were rRNA genes. The plastome was A/T biassed, and the overall GC content was 38.4%. Using maximum likelihood and Bayesian inference methods, the phylogenetic analysis of the complete plastome sequence revealed a close relationship between *C. hystrix* and *C. aurantiifolia*, placing them under the same clade as *C. micrantha*.

## Introduction

**Figure 1.**
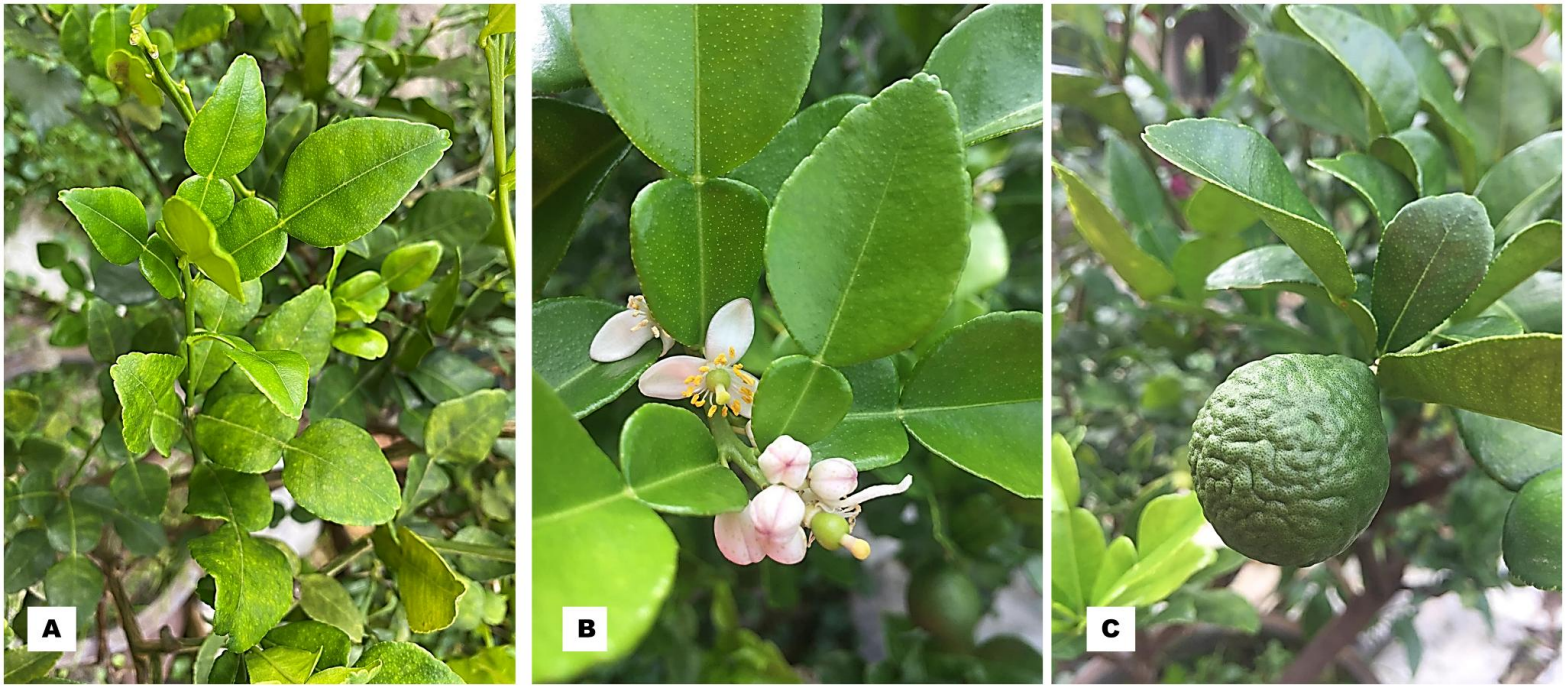
Morphological characteristics of *Citrus hystrix*. (A) distinctive double leaves, (B) small, fragrant, white flower, and (C) large, verrucose, bumpy fruit. Photos by S.Y. Lee.

*Citrus hystrix* DC. 1813, commonly known as kaffir lime or limau purut in Malay, is a member of Rutaceae and is native to tropical Southeast Asia and southern China. The small, bushy tree, typically 3–6 m in height, is known for its thorny branches, distinctive double leaves, and bumpy, acidic fruits (Chandrika Ramadugu et al. 2017; Figure 1). Widely cultivated in suitable climates, it is a staple in Southeast Asian cuisines, especially in Indonesian, Thai, Laotian, Cambodian, and Vietnamese dishes. The leaves, which can be used fresh, dried, or frozen, are particularly valued for their aromatic qualities and are employed in various culinary applications such as soups, curries, and teas. Additionally, traditional medicine across Asia utilizes *C. hystrix* fruit juice and peels for their purported health benefits, incorporating them into shampoos for head lice treatment (Zhao et al. 2023).

Pharmacological research has revealed that *C. hystrix* exhibits numerous medicinal properties, including antimicrobial, antioxidant, anti-tumor, and anti-inflammatory activities (Rahman and Wibowo 2023). These findings align with its traditional uses as an insecticide and treatment for various ailments like heart disease, dizziness, and digestive issues. Some important bioactive compounds in *C. hystrix* are coumarins, flavonoids, phenolic acids, and terpenoids, while bergamottin is a well-known coumarin compound that has a lot of potential in medicine (Abirami et al. 2014).

Genetic studies involving *C. hystrix* have been consistently reported over the years (Gill et al. 2022). However, as an important food and medicinal plant within its region, information on the plastid genome (plastome) sequence is still limited. Thus, in this study, we report on the complete sequence of the plastome of *C. hystrix* and present a phylogenetic analysis of *Citrus* based on the complete plastome genome sequence to understand its molecular placement in the genus.

## Materials and methods

Fresh leaves of *C. hystrix* were collected from the medicinal herb collection in the Traditional Chinese Medicine (TCM) Garden at INTI International University, Nilai, Negeri Sembilan (coordinates: 2˚48′50.4″N, 101˚45′29.52″E). A voucher specimen (collection number: LSY13) has been deposited in the Biotechnology Lab at INTI International University (https://newinti.edu.my, Prof. Dr. Lee Shiou Yih, shiouyih.lee@newinti.edu.my). The specimen collection was approved by the Head of Programme at the Faculty.

Genomic DNA was extracted using the FavorPrepTM Plant Genomic DNA Extraction Mini Kit (Favorgen, Taiwan, China) according to the manufacturer’s instructions. A genomic library with an insert size of approximately 350 bp was prepared using the TruSeq DNA Sample Prep Kit (Illumina, San Diego, CA) and sequenced on the Illumina Novaseq platform (Illumina, CA, USA), generating 150 bp paired-end raw reads. The raw next-generation sequencing (NGS) data was processed and assembled using the NOVOWrap v1.20 pipeline (Wu et al. 2021). The *rbc*L gene sequence of *C. hystrix* (GenBank accession number: MH069764) was used as the seed sequence for the plastome assembly. Genome annotation was performed with GeSeq v2.03 (Tillich et al. 2017) using default parameters, and the results were manually checked for errors. The annotated plastome was visualized using OGDraw 1.3.1 (Greiner et al. 2019), and the structure of genes that are difficult to annotate, including cis-splicing and trans-splicing genes, was verified with CPGView (Liu et al. 2023).

Based on the availability of the plastome data, a total of 30 *Citrus* species were included in the phylogenetic analysis. Commercial hybrids were excluded from the analysis. Two closely related species, *Ruta chalepensis* (GenBank accession number: ON641291; unpublished) and *Zanthoxylum pinnatum* (GenBank accession number: MN968553; Reichelt et al. 2021), were included as outgroups.

The phylogenetic tree was reconstructed using both maximum likelihood (ML) and Bayesian inference (BI) methods, employing the RAxML v8 (Stamatakis, 2014) and MrBayes v3.2 (Ronquist et al. 2012) pipelines available through the CIPRES Science Gateway (Miller et al. 2010). For the ML analysis, the general-time reversible (GTR) model with gamma distribution (+G) was selected, with 1,000 bootstrap replicates. For the BI analysis, a 4 by 4 substitution model and a mixed nucleotide model were chosen, and Markov chain Monte Carlo (MCMC) was performed over 2,000,000 generations, with sampling every 100 cycles. The results were visualized using FigTree v1.4.4 (http://tree.bio.ed.ac.uk/software/figtree/).

## Results

**Figure 2.**
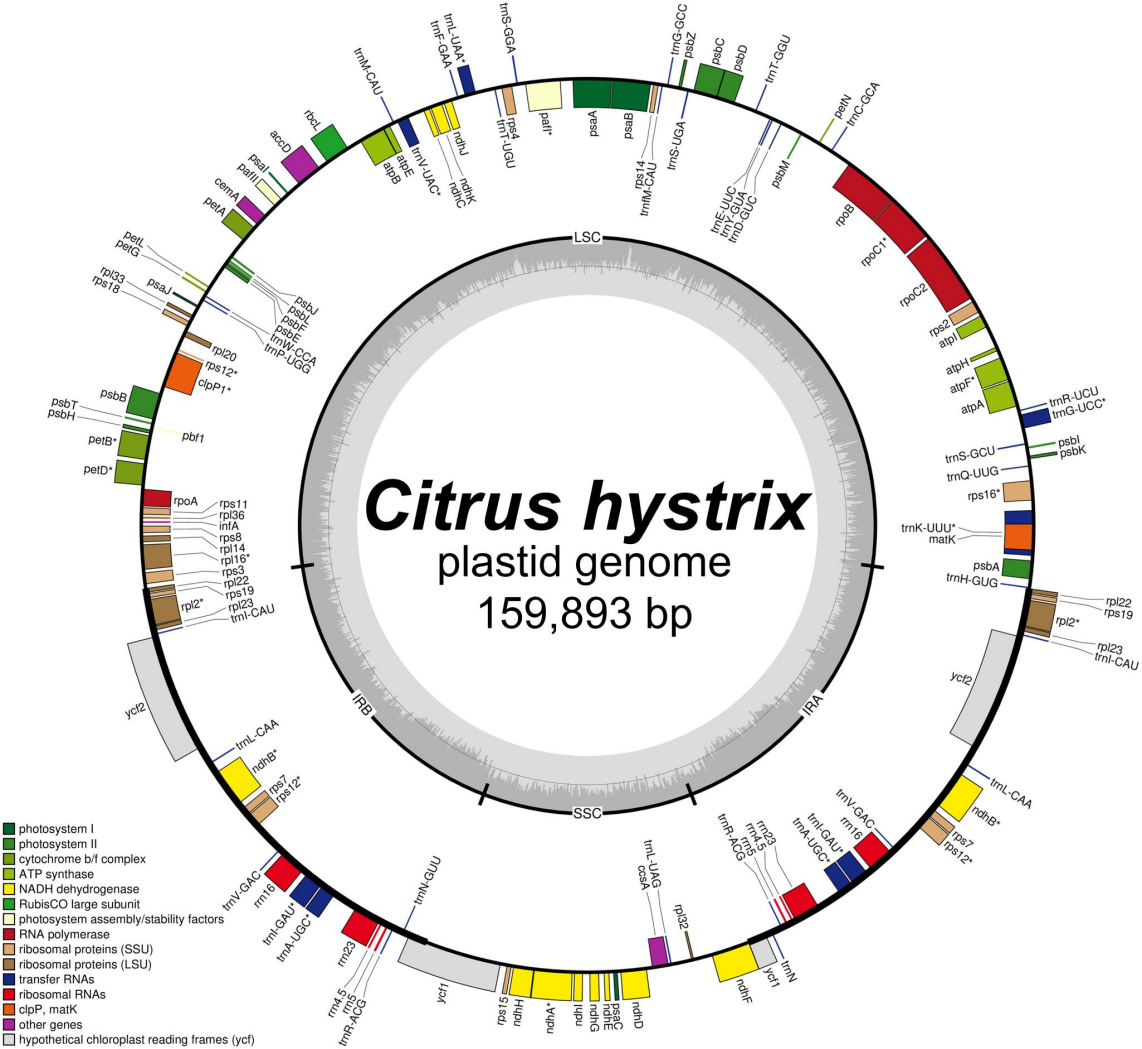
Plastid genome map of *Citrus hystrix*. Genes on inside of map are transcribed in clockwise direction; genes on outside of map are transcribed in counter clockwise direction.

With a minimum read mapping depth of 357× and an average read mapping depth of 1000.4× (Supplementary Figure 1), the complete plastome of *C. hystrix* (GenBank accession number: PQ149287) was determined to be 159,893 bp in size. The plastome exhibited a typical quadripartite structure, consisting of a large single-copy (LSC) region of 87,148 bp and a small single-copy (SSC) region of 18,763 bp, separated by a pair of inverted repeat (IR) regions each 26,991 bp in length (Figure 2). A total of 132 genes were predicted, including 87 protein-coding genes (CDS), 37 tRNA genes, and eight rRNA genes. Among these, 13 CDS were cis-splicing genes, with two containing two introns and 11 containing one intron each (Supplementary Figure 2A). The structure of the trans-splicing gene *rps*12 was also identified (Supplementary Figure 2B). The plastome was found to be A/T-biassed, with base compositions of 48,740 bp for A, 31,117 bp for C, 30,345 bp for G, and 49,691 bp for T. The overall GC content was 38.4%.

**Figure 3.**
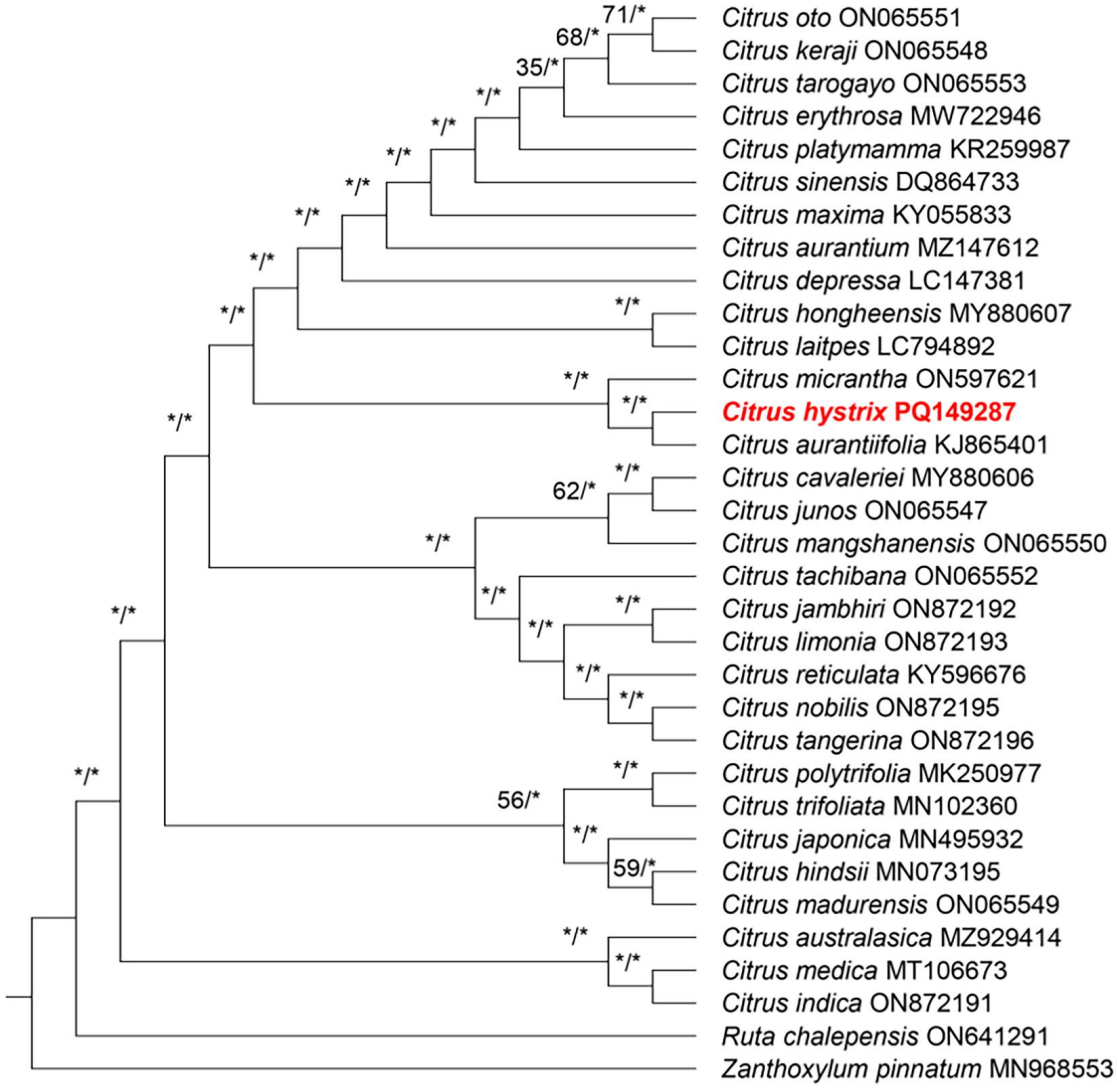
Phylogenetic tree based on the plastid genome sequence of 31 selected species of *Citrus*, with *Ruta chalepensis* (GenBank accession number: ON641291; unpublished); *Zanthoxylum pinnatum* (GenBank accession number: MN968553; Reichelt et al. 2021) included as outgroup. For the maximum likelihood, the GTR+G substitution model was employed, and branch supports were calculated under 1,000 bootstrap replicates; for the Bayesian inference, a 4-by-4 nucleotide model was applied, and MCMC was performed under 2,000,000 generations with sampling taken at every 100 cycles. The bootstrap support (BS; left) and posterior probability (PP; right) values are indicated at each branch node, of which BS ≥ 75% and PP ≥ 0.95 are indicated with an asterisk (*). The following sequences were used: *C. aurantiifolia* (GenBank accession number: KJ865401; Su et al. 2014), *C. aurantium* (GenBank accession number: MZ147612; unpublished), *C. australasica* (GenBank accession number: MZ929414; Cai et al. 2022), *C. cavaleriei* (GenBank accession number: MT880606; Zhang et al. 2020a), *C. depressa* (GenBank accession number: LC147381; Ishikawa et al. 2016), *C. erythrosa* (GenBank accession number: MW722946; Shin et al. 2022), *C. hindsii* (GenBank accession number: MN073195; Xu et al. 2019), *C. hystrix* (GenBank accession number: PQ149287; this study), *C. hongheensis* (GenBank accession number: MT880607; Zhang et al. 2020b), *C. indica* (GeneBank accession number: ON872191; Shi et al. 2023), *C. jambhiri* (GenBank accession number: ON872192; Shi et al. 2023), *C. japonica* (GenBank accession number: MN495932), *C. junos* (GenBank accession number: ON065547; Shi et al. 2023), *C. keraji* (GenBank accession number: ON065548; Shi et al. 2023), *C. latipes* (GenBank accession number: LC794892), *C. limonia* (GenBank accession number: ON872193; Shi et al. 2023), *C. madurensis* (GenBank accession number: ON065549; Shi et al. 2023), *C. mangshanensis* (GenBank accession number: ON065550; Shi et al. 2023), *C. maxima* (GenBank accession number: KY055833; Liu and Shi 2017), *C. medica* (GenBank accession number: MT106673; Zhang and Bai 2020), *C. micrantha* (GenBank accession number: ON597621; Madayag et al. 2022), *C. nobilis* (GenBank accession number: ON872195; Shi et al. 2023), *C. oto* (GenBank accession number: ON065551; Shi et al. 2023), *C. platymamma* (GenBank accession number: KR259987; Lee et al. 2015), *C. polytrifolia* (GenBank accession number: MK250977; Li et al. 2019), *C. reticulata* (GenBank accession number: KY596676; unpublished), *C. sinensis* (GenBank accession number: DQ864733; Bausher et al. 2006), *C. tachibana* (GenBank accession number: ON065552; Shi et al. 2023), *C. tangerina* (GenBank accession number: ON872196; Shi et al. 2023), *C. tarogayo* (GenBank accession number: ON065548; Shi et al. 2023), and *C. trifoliata* (GenBank accession number: MN102360; He et al. 2020).

Both the ML and BI trees showed the same topology; as a result, the trees were combined and only the ML tree was shown. The phylogenetic relationship within *Citrus* is mostly resolved (BS ≥75%, PP ≥ 0.95), except for the split between the *C. hindsii*+*C. japonica*+*C. madurensis* clade and *C. polytrifolia*+*C. trifoliata* clade (BS = 56%), the split between *C. hindsii* and *C. madurensis* (BS = 59%), the split for *C. mangshanensis* (BS = 62%), the split for *C. erythorosa + C. keraji + C. oto + C. platymamma + C. sinensis + C. tarogaya* clade (BS = 71%), and the split for *C. aurantium* (BS = 68%). *Citrus hystrix* is closely related to *C. aurantiifolia* and is placed under the same clade as *C. micrantha*.

## Discussion

This is the first report on the complete plastome sequence of *C. hystrix*. The plastome of *C. hystrix* shared the same gene structure as other published *Citrus* species that are publicly available (Shi et al. 2023). The genome size and gene content are similar to those of other *Citrus* species, i.e. *C. aurantiifolia* (Christm.) Swingle (Su et al. 2014). For the phylogenetic analysis, the low branch support present in the tree could be due to the limited sampling size; an increased sampling size could reduce phylogenetic error (Zwickl and Hillis, 2002). For the molecular placement of *C. hystrix*, based on the complete plastome sequence, *C. hystrix* is placed under the Micrantha clade, as proposed by Nicolosi et al. (2000). Genome sequence alignment revealed that there are only 14 indels present between the complete plastome sequences of *C. aurantiifolia* and *C. hystrix*, of which the former is a natural hybrid species between *C. micrantha* Wester (female parent) and *C. medica* L. (male parent) (Rouiss et al. 2018). It is also proposed that *C. micrantha* is a synonym for *C. hystrix* (Mabberley 2022), which explains the high similarity in the plastome sequence of *C. aurantiifolia* when compared to *C. hystrix*.

## Supplementary Material

FigS2_new.tiff

FigS1.tiff

figs3.tif

clean_lee et al_mitob_202401216.docx

## Data Availability

The genome sequence data that support the findings of this study are openly available in GenBank of NCBI at http://www.ncbi.nlm.nih.gov under the accession number PQ149287. The associated BioProject, SRA, and BioSample numbers are PRJNA853926, SRR30119633, and SAMN43020313, respectively.

## Permission for sample collection

*Citrus hystrix* is not a protected plant. Permission for sample collection at the TCM Garden of INTI International University is acknowledged by the Head of Program of the Faculty.
