# Supplementary figures and images for: The complete plastid genome of Citrus hystrix DC. 1813 (Rutaceae) and its phylogenetic analysis
Source: Mitochondrial DNA B Resour. 2025 Jan 7;10(1):94–8. doi: 10.1080/23802359.2025.2449723 (PMC11721868; doi:10.1080/23802359.2025.2449723)

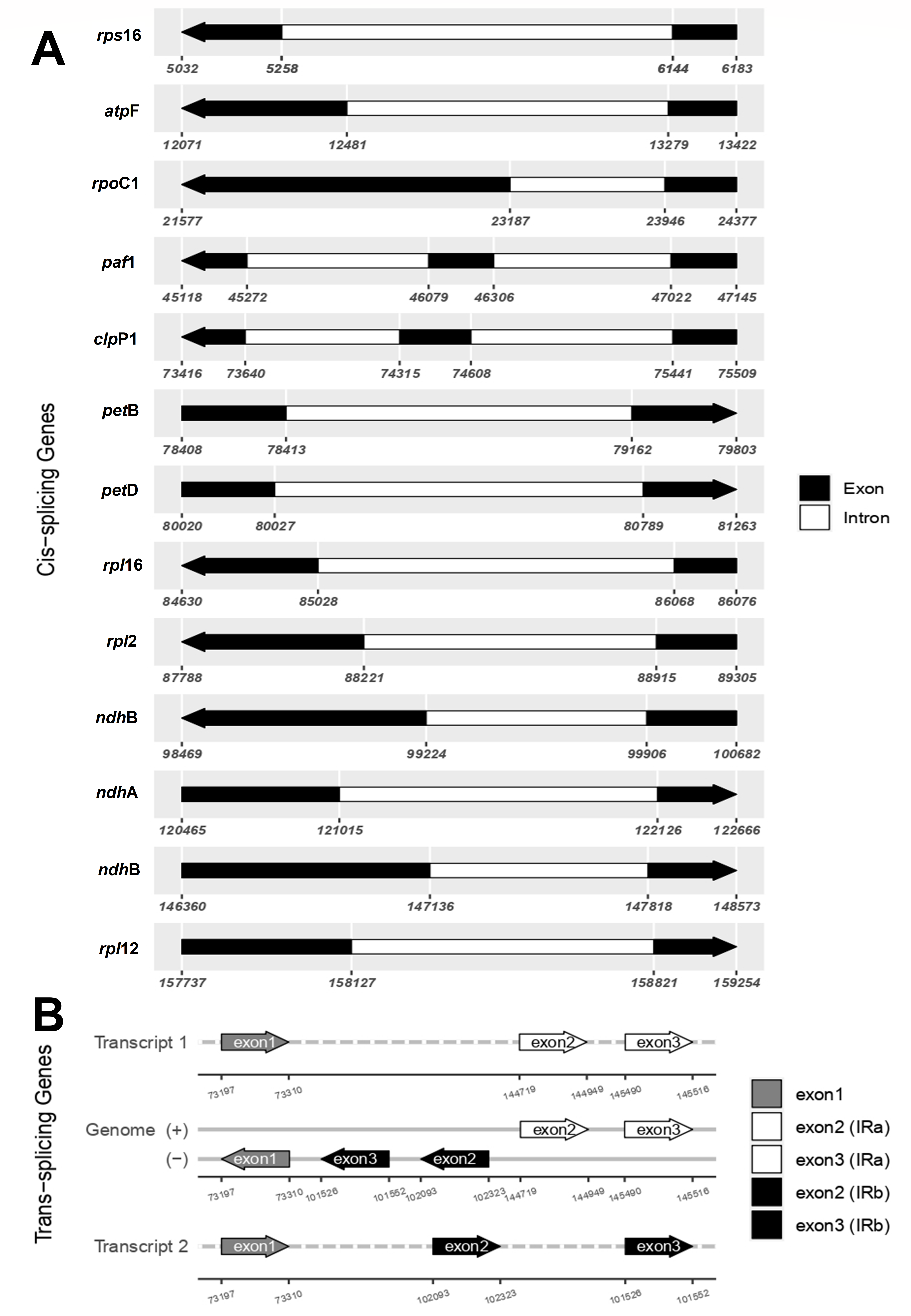

Supplement: FigS2_new.tiff [file TMDN_A_2449723_SM6883.tiff]

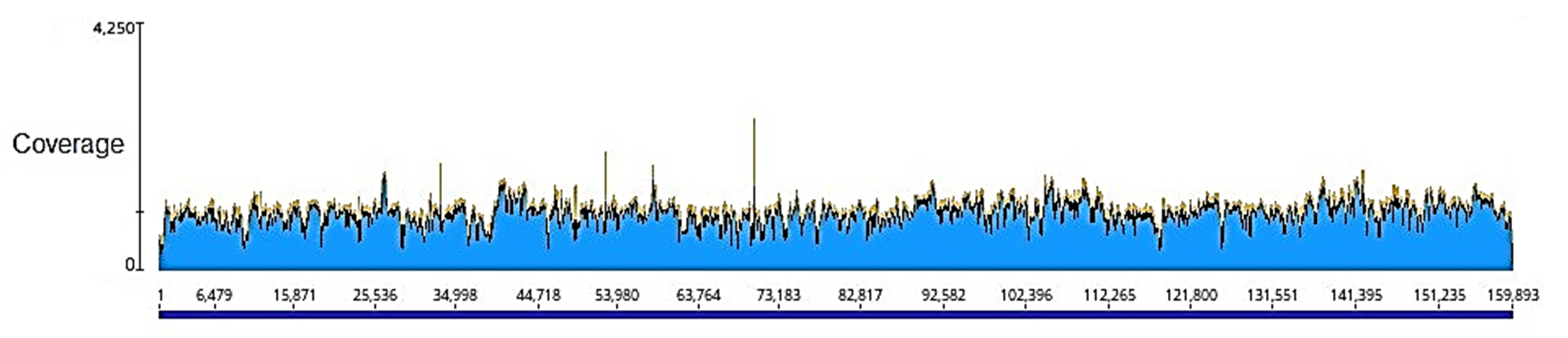

Supplement: FigS1.tiff [file TMDN_A_2449723_SM6882.tiff]

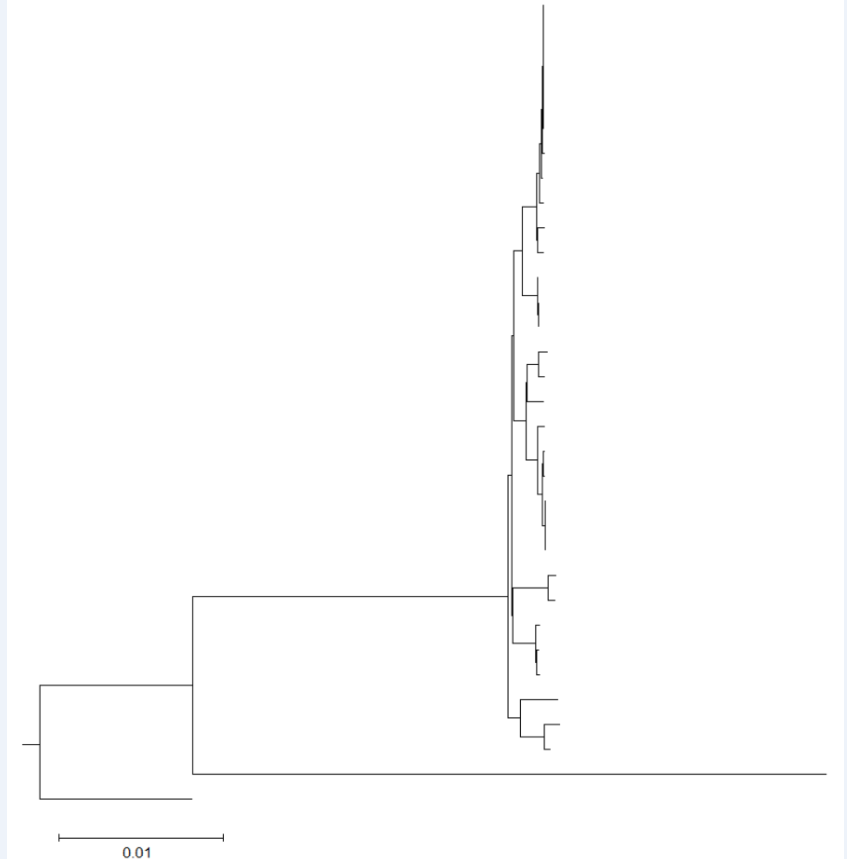

Supplement: figs3.tif [file TMDN_A_2449723_SM6881.tif]
