# Supplementary material for: The complete plastid genome of Citrus hystrix DC. 1813 (Rutaceae) and its phylogenetic analysis
Source: Mitochondrial DNA B Resour. 2025 Jan 7;10(1):94–8. doi: 10.1080/23802359.2025.2449723 (PMC11721868; doi:10.1080/23802359.2025.2449723)
Supplement: clean_lee et al_mitob_202401216.docx [file TMDN_A_2449723_SM6880.docx]

**The complete plastid genome of *Citrus hystrix* DC. 1813 (Rutaceae) and its phylogenetic analysis**

Wan Shuan Lee^a,e^, Warut Donrung^b^, Bui Manh Hung^c^, Nurien Hidayu Muhamad Rusly^a^, Shiou Yih Lee^d,e^*, Tawatchai Tanee^b,f^*

*^a^Faculty of Education and Liberal Arts, INTI International University, Nilai, Negeri Sembilan, Malaysia*

*^b^Faculty of Environment and Resource Studies, Mahasarakham University, 44150 Maha Sarakham, Thailand*

*^c^Department of Forest Inventory and Planning, Faculty of Forestry, Vietnam National University of Forestry, Chuong My, Hanoi, Vietnam*

*^d^Faculty of Health and Life Sciences, INTI International University, Nilai, Negeri Sembilan, Malaysia*

*^e^Centre of Health, Well-Being, and Environmental Sustainability, INTI International University, Nilai, Negeri Sembilan, Malayisa*

*^f^One Health Research Unit, Mahasarakham University, Maha Sarakham, Thailand*

Corresponding author

Name: Shiou Yih Lee

Affiliation: Faculty of Health and Life Sciences, INTI International University, 71800 Nilai, Negeri Sembilan, Malaysia

E-mail: shiouyih.lee@newinti.edu.my

Name: Tawatchai Tanee

Affiliation: Faculty of Environment and Resource Studies, Mahasarakham University, 44150 Maha Sarakham, Thailand

E-mail: tawatchai5@hotmail.com

**Abstract**

*Citrus hystrix* DC. 1813, also known as kaffir lime, is a widely cultivated *Citrus* species in the Southeast Asia region that is used for flavour, fragrance, and medicine. However, research on *C. hystrix* is limited at present. To provide useful genetic information on this valuable plant species, in this study, the plastid genome (plastome) of *C. hystrix* was characterised and its phylogenetic position was investigated. The complete plastome size of *C. hystrix* is 159,893 bp in length and has a typical quadripartite structure. The 87,148-bp-long large single-copy and the 18,763-bp-long small single-copy regions were separated by a pair of inverted repeats (each 26,991 bp). The plastome was predicted to contain 132 genes, of which 87 were CDS, 37 were tRNA, and eight were rRNA genes. The plastome was A/T biassed, and the overall GC content was 38.4%. Using maximum likelihood and Bayesian inference methods, the phylogenetic analysis of the complete plastome sequence revealed a close relationship between *C. hystrix* and *C. aurantiifolia*, placing them under the same clade as *C. micrantha*. The data provided will be useful for the study of the molecular phylogenetics and evolution of *Citrus* in the future.

Keywords: Aurantidioideae, *Citrus micrantha*, genetic resources, kaffir lime, limau purut

**Introduction**

*Citrus hystrix* DC. 1813, commonly known as kaffir lime or limau purut in Malay, is a member of Rutaceae and is native to tropical Southeast Asia and southern China. The small, bushy tree, typically 3-6 m in height, is known for its thorny branches, distinctive double leaves, and bumpy, acidic fruits (Chandrika Ramadugu et al., 2017; Figure 1). Widely cultivated in suitable climates, it is a staple in Southeast Asian cuisines, especially in Indonesian, Thai, Laotian, Cambodian, and Vietnamese dishes. The leaves, which can be used fresh, dried, or frozen, are particularly valued for their aromatic qualities and are employed in various culinary applications such as soups, curries, and teas. Additionally, traditional medicine across Asia utilises *C. hystrix* fruit juice and peels for their purported health benefits, incorporating them into shampoos for head lice treatment (Zhao et al., 2023).

Pharmacological research has revealed that *C. hystrix* exhibits numerous medicinal properties, including antimicrobial, antioxidant, anti-tumour, and anti-inflammatory activities (Rahman and Wibowo, 2023). These findings align with its traditional uses as an insecticide and treatment for various ailments like heart disease, dizziness, and digestive issues. Some important bioactive compounds in *C. hystrix* are coumarins, flavonoids, phenolic acids, and terpenoids, while bergamottin is a well-known coumarin compound that has a lot of potential in medicine (Abirami et al., 2014).

Genetic studies involving *C. hystrix* has been consistently reported over the years (Gill et al., 2022). However, as an important food and medicinal plant within its region, information on the plastid genome (plastome) sequence is still limited. Thus, in this study, we report on the complete sequence of the plastome of *C. hystrix* and present a phylogenetic analysis of *Citrus* based on the complete plastome genome sequence to understand its molecular placement in the genus.

**Materials and Methods**

Fresh leaves of *C. hystrix* were collected from the medicinal herb collection in the Traditional Chinese Medicine (TCM) Garden at INTI International University, Nilai, Negeri Sembilan (coordinates: 2˚48′50.4″N, 101˚45′29.52″E). A voucher specimen (collection number: LSY13) has been deposited in the Biotechnology Lab at INTI International University (<https://newinti.edu.my>, Prof. Dr. Lee Shiou Yih, shiouyih.lee@newinti.edu.my). The specimen collection was approved by the Head of Programme at the Faculty.

Genomic DNA was extracted using the FavorPrepTM Plant Genomic DNA Extraction Mini Kit (Favorgen, Taiwan, China) according to the manufacturer’s instructions. A genomic library with an insert size of approximately 350 bp was prepared using the TruSeq DNA Sample Prep Kit (Illumina, San Diego, CA) and sequenced on the Illumina Novaseq platform (Illumina, CA, USA), generating 150 bp paired-end raw reads. The raw next-generation sequencing (NGS) data was processed and assembled using the NOVOWrap v1.20 pipeline (Wu et al., 2021). The *rbc*L gene sequence of *C. hystrix* (GenBank accession number: MH069764) was used as the seed sequence for the plastome assembly. Genome annotation was performed with GeSeq v2.03 (Tillich et al. 2017) using default parameters, and the results were manually checked for errors. The annotated plastome was visualised using OGDraw 1.3.1 (Greiner et al. 2019), and the structure of genes that are difficult to annotate, including cis-splicing and trans-splicing genes, was verified with CPGView (Liu et al. 2023).

Based on the availability of the plastome data, a total of 30 *Citrus* species were included in the phylogenetic analysis. Commercial hybrids were excluded from the analysis. Two closely related species, *Ruta chalepensis* (GenBank accession number: ON641291; unpublished) and *Zanthoxylum pinnatum* (GenBank accession number: MN968553; Reichelt et al. 2021), were included as outgroups.

The phylogenetic tree was reconstructed using both maximum likelihood (ML) and Bayesian inference (BI) methods, employing the RAxML v8 (Stamatakis, 2014) and MrBayes v3.2 (Ronquist et al., 2012) pipelines available through the CIPRES Science Gateway (Miller et al., 2010). For the ML analysis, the general-time reversible (GTR) model with gamma distribution (+G) was selected, with 1,000 bootstrap replicates. For the BI analysis, a 4 by 4 substitution model and a mixed nucleotide model were chosen, and Markov chain Monte Carlo (MCMC) was performed over 2,000,000 generations, with sampling every 100 cycles. The results were visualised using FigTree v1.4.4 (http://tree.bio.ed.ac.uk/software/figtree/).

**Results**

With a minimum read mapping depth of 357× and an average read mapping depth of 1000.4× (Supplementary Figure 1), the complete plastome of C. hystrix (GenBank accession number: PQ149287) was determined to be 159,893 bp in size. The plastome exhibited a typical quadripartite structure, consisting of a large single-copy (LSC) region of 87,148 bp and a small single-copy (SSC) region of 18,763 bp, separated by a pair of inverted repeat (IR) regions each 26,991 bp in length (Figure 2). A total of 132 genes were predicted, including 87 protein-coding genes (CDS), 37 tRNA genes, and eight rRNA genes. Among these, 13 CDS were cis-splicing genes, with two containing two introns and 11 containing one intron each (Supplementary Figure 2A). The structure of the trans-splicing gene rps12 was also identified (Supplementary Figure 2B). The plastome was found to be A/T-biassed, with base compositions of 48,740 bp for A, 31,117 bp for C, 30,345 bp for G, and 49,691 bp for T. The overall GC content was 38.4%.

Both the ML and BI trees showed the same topology; as a result, the trees were combined and only the ML tree was shown. The phylogenetic relationship within *Citrus* is mostly resolved (BS ≥75%, PP ≥ 0.95), except for the split between the *C. hindsii*+*C. japonica*+*C. madurensis* clade and *C. polytrifolia*+*C. trifoliata* clade (BS = 56%), the split between *C. hindsii* and *C. madurensis* (BS = 59%), the split for *C. mangshanensis* (BS = 62%), the split for *C. erythorosa+C. keraji+C. oto+C. platymamma+C. sinensis+C. tarogaya* clade (BS = 71%), and the split for *C. aurantium* (BS = 68%). *Citrus hystrix* is closely related to *C. aurantiifolia* and is placed under the same clade as *C. micrantha*.

**Discussion**

This is the first report on the complete plastome sequence of *C. hystrix*. The plastome of *C. hystrix* shared the same gene structure as other published *Citrus* species that are publicly available (Shi et al., 2023). The genome size and gene content are similar to those of other *Citrus* species, i.e., *C. aurantiifolia* (Christm.) Swingle (Su et al., 2014). For the phylogenetic analysis, the low branch support present in the tree could be due to the limited sampling size; an increased sampling size could reduce phylogenetic error (Zwickl and Hillis, 2022). For the molecular placement of *C. hystrix*, based on the complete plastome sequence, *C. hystrix* is placed under the Micrantha clade, as proposed by Nicolosi et al. (2000). Genome sequence alignment revealed that there are only 14 indels present between the complete plastome sequences of *C. aurantiifolia* and *C. hystrix*, of which the former is a natural hybrid species between *C. micrantha* Wester (female parent) and *C. medica* L. (male parent) (Rouiss et al., 2018). It is also proposed that *C. micrantha* is a synonym for *C. hystrix* (Mabberley, 2022), which explains the high similarity in the plastome sequence of *C. aurantiifolia* when compared to *C. hystrix*.

**Supplemental material**

Supplementary Figure 1. Read mapping depth (blue region) of the plastome sequence generated in this study. Y-axis shows the read mapping depth, while X-axis indicates the nucleotide position of the plastome.

Supplementary Figure 2. Structure of the genes that are difficult to annotate. (a) cis-splicing genes, and (b) trans-splicing genes.

Supplementary Figure 3. Phylogram for the phylogenetic tree showed in Figure 3.

**Disclosure statement**

The authors report no conflict of interest.

**Permission for sample collection**

*Citrus hystrix* is not a protected plant. Permission for sample collection at the TCM Garden of INTI International University is acknowledged by the Head of Program of the Faculty.

**Funding**

This study was supported by Mahasarakham University and the INTI IU Research Seeding Grant under Grant [number INTI-FHLS-02-16-2023].

**Data availability statement**

The genome sequence data that support the findings of this study are openly available in GenBank of NCBI at http://www.ncbi.nlm.nih.gov under the accession number PQ149287. The associated BioProject, SRA, and BioSample numbers are PRJNA853926, SRR30119633, and SAMN43020313, respectively.

**Author contribution statement**

BMH, NHMR, SYL, TT: conception and design; WSL, WD: analysis and interpretation of data; WSL, WD: drafting of the paper; BMH, NHMR, SYL, TT: critical revision of the paper; all the authors approved the final version; and all authors agree to be accountable for all aspects of the work.

**References**

Abirami A, Nagarani G, Siddhuraju P. 2014. The medicinal and nutritional role of underutilized citrus fruit *Citrus hystrix* (Kaffir lime): A review. Drug Invent. Today. 6(1): 1-5.

Bausher MG, Singh ND, Lee SB, Jansen RK, Daniell H. 2006. The complete chloroplast genome sequence of *Citrus sinensis* (L.) Osbeck var ‘Ridge Pineapple’: organization and phylogenetic relationships to other angiosperms. BMC Plant Biol. 6: 21.

Cai QN, Wang HX, Chen DJ, Ke XR, Zhu ZX, Wang HF. 2022. The complete chloroplast genome sequence of a *Citrus australasica* cultivar (Rutaceae). Mitochondrial DNA B Resour. 7(1): 54-55.

Chandrika Ramadugu CR, Razi MF, Keremane ML, Scora RW, Roose ML. 2017. Systematic classification, distribution and botany. In MM Khan, R Al-Yahyai, F Al-Said (eds.) The lime: botany, production and uses. Wallingford UK: CABI; p. 12-36.

Gill K, Kumar P, Kumar A, Kapoor B, Sharma R, Joshi AK. 2022. Comprehensive mechanistic insights into the citrus genetics, breeding challenges, biotechnological implications, and omics-based interventions. Tree Genet. Genomes. 18(2): 9.

Greiner S, Lehwark P, Bock R. 2019. OrganellarGenomeDRAW (OGDRAW) version 1.3.1: expanded toolkit for the graphical visualization of organellar genomes. Nucleic Acids Res. 47(W1): W59-W64.

He SL, Tian Y, Yang Y, Shi CY. 2020. Chloroplast genome and phylogenetic analyses of *Poncirus trifoliata* (Rutaceae). Mitochondrial DNA B Resour. 5(3): 2205-2206.

Ishikawa R, Badenoch N, Miyagi K, Medoruma K, Osada T, Onishi M. 2016. Multi-lineages of Shiikuwasha (*Citrus depressa* Hayata) evaluated by using whole chloroplast genome sequences and its bio-diversity in Okinawa, Japan. Breed. Sci. 66(4): 490-498.

Lee M, Park J, Lee H, Sohn SH, Lee J. 2015. Complete chloroplast genomic sequence of *Citrus platymamma* determined by combined analysis of Sanger and NGS data. Hortic. Environ. Biote. 56: 704-711.

Li S, Zong D, Zhou A, He C. 2019. The complete chloroplast genome sequence of *Poncirus polyandra* (Rutaceae), an endangered species endemic to Yunnan Province, China. Mitochondrial DNA B Resour. 4(1): 766-768.

Liu J, Shi C. 2017. The complete chloroplast genome of wild shaddock, *Citrus maxima* (Burm.) Merr. Conserv. Genet. Resour. 9(4): 599-601.

Liu S, Ni Y, Li J, Zhang X, Yang H, Chen H, Liu C. 2023. CPGView: a package for visualizing detailed chloroplast genome structures. Mol. Ecol. Resour. 23(3): 694-704.

Mabberley DJ. 2022. A classification for edible citrus: an update, with a note on Murraya (Rutaceae). Telopea. 25: 271-284.

Madayag RE, Gentallan RJP, Quiñones KJO, Bartolome MCB, Vera Cruz JRA, Borromeo TH, Endonela LE, Timog EBS. 2022. The complete chloroplast genome of ‘biasong’ (*Citrus micrantha* Wester), a native species from the Southern Philippines. Mitochondrial DNA B Resour. 7(11): 1992-1996.

Miller MA, Pfeiffer W, Schwartz T. 2010. Creating the CIPRES science gateway for inference of large phylogenetic trees. New Orleans: Proceedings of the Gateway Computing Environments Workshop (GCE); p. 1–8.

Nicolosi E, Deng ZN, Gentile A, La Malfa S, Continella G, Tribulato E. 2000. *Citrus* phylogeny and genetic origin of important species as investigated by molecular markers. Theor. Appl. Genet. 100: 1155-1166.

Rahman PK, Wibowo TS. 2023. Pharmacological activities of *Citrus hystrix*. Indones. J. Interdiscip. Res. Sci. Technol. 1(7): 641-650.

Reichelt N, Wen J, Pätzold C, Appelhans MS. 2021. Characterization of the complete chloroplast genome sequences of four *Zanthoxylum* L. species (Sapindales: Rutaceae) from the Caribbean, Madagascar, the Mascarene Islands, and the South Pacific. Microbiol. Resour. Announc. 10: e00399-21.

Ronquist F, Teslenko M, van der Mark P, Ayres DL, Darling A, Höhna S, Larget B, Liu L, Suchard MA, Huelsenbeck JP. 2012. MrBayes 3.2: efficient Bayesian phylogenetic inference and model choice across a large model space. Syst Biol. 61(3): 539–542.

Rouiss H, Bakry F, Froelicher Y, Navarro L, Aleza P, Ollitrault P. 2018. Origin of *C. latifolia* and *C. aurantiifolia* triploid limes: the preferential disomic inheritance of doubled-diploid ‘Mexican’lime is consistent with an interploid hybridization hypothesis. Ann. Bot. 121(3): 571-585.

Shi W, Song W, Liu J, Shi C, Wang S. 2023. Comparative chloroplast genome analysis of *Citrus* (Rutaceae) species: Insights into genomic characterization, phylogenetic relationships, and discrimination of subgenera. Sci. Hortic. 313: 111909.

Shin SC, Song JH, Yoo YH, Lee JS, Kang SI, Kim HJ, Lee H, Kim HB. 2022. The complete chloroplast genome sequence of a medicinal citrus landrace, *Citrus erythrosa* Hort. ex Tanaka in Jeju Island, Korea. Mitochondrial DNA B Resour. 7(4): 580-582.

Su HJ, Hogenhout SA, Al-Sadi AM, Kuo CH. 2014. Complete chloroplast genome sequence of Omani lime (*Citrus aurantiifolia*) and comparative analysis within the rosids. PloS One. 9(11): e113049.

Tillich M, Lehwark P, Pellizzer T, Ulbricht-Jones ES, Fischer A, Bock R, Greiner S. 2017. GeSeq – versatile and accurate annotation of organelle genomes. Nucleic Acids Res. 45(W1): W6-W11.

Wu P, Xu C, Chen H, Yang J, Zhang X, Zhou S. 2021. NOVOWrap: an automated solution for plastid genome assembly and structure standardization. Mol. Ecol. Resour. 21(6): 2177-2186.

Xu SR, Zhang YY, Liu F, Tian N, Pan DM, Bei XJ, Cheng CZ. 2019. Characterization of the complete chloroplast genome of the Hongkong kumquat (*Fortunella hindsii* Swingle). Mitochondrial DNA B Resour. 4(2): 2612-2613.

Zhang F, Bai D. 2020. The complete chloroplast genome of *Citrus medica* (Rutaceae). Mitochondrial DNA B Resour. 5(2): 1627-1629.

Zhang ZH, Long CR, Jiang Y, Bei XJ, Wang SH. 2020b. Characterization of the complete chloroplast genome of *Citrus hongheensis*, a key protected wild plant in Yunnan province of China. Mitochondrial DNA B Resour. 5(3): 3514-3515.

Zhang ZH, Long CR, Jiang Y, Yang SZ, Zhao J, Wang SH. 2020a. Characterization of the complete chloroplast genome of Yuanjiang wild Ichang papeda (*Citrus cavaleriei*) in China. Mitochondrial DNA B Resour. 5(3): 3349-3350.

Zhao Z, Wang Y, Nian M, Lv H, Chen J, Qiao H, Yang X, Li X, Chen X, Zheng X, Wu S. 2023. *Citrus hystrix*: a review of phytochemistry, pharmacology and industrial applications research progress. Arab. J. Chem. 16(11): 105236.

Zwickl DJ, Hillis DM. 2002. Increased taxon sampling greatly reduces phylogenetic error. System. Biol. 51(4): 588-598.

**Figure captions**

**Figure 1.** Morphological characteristics of *Citrus hystrix*. (A) distinctive double leaves, (B) small, fragrant, white flower, and (C) large, verrucose, bumpy fruit. Photos by S.Y. Lee.

**Figure 2.** Plastid genome map of *Citrus hystrix*. Genes on inside of map are transcribed in clockwise direction; genes on outside of map are transcribed in counter clockwise direction.

**Figure 3.** Phylogenetic tree based on the plastid genome sequence of 31 selected species of *Citrus*, with *Ruta chalepensis* (GenBank accession number: ON641291; unpublished); *Zanthoxylum pinnatum* (GenBank accession number: MN968553; Reichelt et al. 2021) included as outgroup. For the maximum likelihood, the GTR+G substitution model was employed, and branch supports were calculated under 1,000 bootstrap replicates; for the Bayesian inference, a 4-by-4 nucleotide model was applied, and MCMC was performed under 2,000,000 generations with sampling taken at every 100 cycles. The bootstrap support (BS; left) and posterior probability (PP; right) values are indicated at each branch node, of which BS ≥ 75% and PP ≥ 0.95 are indicated with an asterisk (*). The following sequences were used: *C. aurantiifolia* (GenBank accession number: KJ865401; Su et al., 2014), *C. aurantium* (GenBank accession number: MZ147612; unpublished), *C. australasica* (GenBank accession number: MZ929414; Cai et al., 2022), *C. cavaleriei* (GenBank accession number: MT880606; Zhang et al., 2020a), *C. depressa* (GenBank accession number: LC147381; Ishikawa et al., 2016), *C. erythrosa* (GenBank accession number: MW722946; Shin et al., 2022), *C. hindsii* (GenBank accession number: MN073195; Xu et al., 2019), *C. hystrix* (GenBank accession number: PQ149287; this study), *C. hongheensis* (GenBank accession number: MT880607; Zhang et al., 2020b), *C. indica* (GeneBank accession number: ON872191; Shi et al., 2023), *C. jambhiri* (GenBank accession number: ON872192; Shi et al., 2023), *C. japonica* (GenBank accession number: MN495932), *C. junos* (GenBank accession number: ON065547; Shi et al., 2023), *C. keraji* (GenBank accession number: ON065548; Shi et al., 2023), *C. latipes* (GenBank accession number: LC794892), *C. limonia* (GenBank accession number: ON872193; Shi et al., 2023), *C. madurensis* (GenBank accession number: ON065549; Shi et al., 2023), *C. mangshanensis* (GenBank accession number: ON065550; Shi et al., 2023), *C. maxima* (GenBank accession number: KY055833; Liu and Shi, 2017), *C. medica* (GenBank accession number: MT106673; Zhang and Bai, 2020), *C. micrantha* (GenBank accession number: ON597621; Madayag et al., 2022), *C. nobilis* (GenBank accession number: ON872195; Shi et al., 2023), *C. oto* (GenBank accession number: ON065551; Shi et al., 2023), *C. platymamma* (GenBank accession number: KR259987; Lee et al., 2015), *C. polytrifolia* (GenBank accession number: MK250977; Li et al., 2019), *C. reticulata* (GenBank accession number: KY596676; unpublished), *C. sinensis* (GenBank accession number: DQ864733; Bausher et al., 2006), *C. tachibana* (GenBank accession number: ON065552; Shi et al., 2023), *C. tangerina* (GenBank accession number: ON872196; Shi et al., 2023), *C. tarogayo* (GenBank accession number: ON065548; Shi et al., 2023), and *C. trifoliata* (GenBank accession number: MN102360; He et al., 2020).
